# Comparing the effects of four common drug classes on the progression of mild cognitive impairment to dementia using electronic health records

**DOI:** 10.1038/s41598-023-35258-6

**Published:** 2023-05-19

**Authors:** Jie Xu, Fei Wang, Chengxi Zang, Hao Zhang, Kellyann Niotis, Ava L. Liberman, Cynthia M. Stonnington, Makoto Ishii, Prakash Adekkanattu, Yuan Luo, Chengsheng Mao, Luke V. Rasmussen, Zhenxing Xu, Pascal Brandt, Jennifer A. Pacheco, Yifan Peng, Guoqian Jiang, Richard Isaacson, Jyotishman Pathak

**Affiliations:** 1 University of Florida, Gainesville, FL USA; 2 Weill Cornell Medicine, New York, NY USA; 3 Mayo Clinic, Scottsdale, AZ USA; 4 Northwestern University, Chicago, IL USA; 5 University of Washington, Seattle, WA USA; 6 Mayo Clinic, Rochester, MN USA

**Keywords:** Neurological disorders, Drug screening

## Abstract

The objective of this study was to investigate the potential association between the use of four frequently prescribed drug classes, namely antihypertensive drugs, statins, selective serotonin reuptake inhibitors, and proton-pump inhibitors, and the likelihood of disease progression from mild cognitive impairment (MCI) to dementia using electronic health records (EHRs). We conducted a retrospective cohort study using observational EHRs from a cohort of approximately 2 million patients seen at a large, multi-specialty urban academic medical center in New York City, USA between 2008 and 2020 to automatically emulate the randomized controlled trials. For each drug class, two exposure groups were identified based on the prescription orders documented in the EHRs following their MCI diagnosis. During follow-up, we measured drug efficacy based on the incidence of dementia and estimated the average treatment effect (ATE) of various drugs. To ensure the robustness of our findings, we confirmed the ATE estimates via bootstrapping and presented associated 95% confidence intervals (CIs). Our analysis identified 14,269 MCI patients, among whom 2501 (17.5%) progressed to dementia. Using average treatment estimation and bootstrapping confirmation, we observed that drugs including rosuvastatin (ATE = − 0.0140 [− 0.0191, − 0.0088], *p* value < 0.001), citalopram (ATE = − 0.1128 [− 0.125, − 0.1005], *p* value < 0.001), escitalopram (ATE = − 0.0560 [− 0.0615, − 0.0506], *p* value < 0.001), and omeprazole (ATE = − 0.0201 [− 0.0299, − 0.0103], *p* value < 0.001) have a statistically significant association in slowing the progression from MCI to dementia. The findings from this study support the commonly prescribed drugs in altering the progression from MCI to dementia and warrant further investigation.

## Introduction

Dementia is a growing global public health challenge that is expected to increase significantly due to population aging. As of 2018, dementia accounted for about 5% of the total global burden of disease, with a total healthcare and societal cost estimated to be more than $1 trillion. This cost is projected to double by 2030, highlighting the urgent need to address this issue^1,2^. Among all forms of dementia, Alzheimer's disease (AD) is the most common, accounting for 80% of all dementia diagnoses^3^. Dementia is characterized by a gradual and progressive decline in cognition function, including memory, language, problem-solving skills, and other cognitive areas. This decline ultimately results in the loss of ability to perform everyday activities and social functioning and places a significant burden on caregivers and the healthcare system^4^. While the causes of dementia are not fully understood, it is believed to be related to multiple factors, including genetic, environmental, and lifestyle factors^5^. There is an urgent need for disease-modifying therapies for dementia, and research efforts are focused on identifying effective interventions to delay, prevent, or cure dementia.

Drug development for new treatments for dementia, including AD, has been a major research focus in recent years, consuming significant resources. However, despite these efforts, most clinical trials for dementia treatments have not yielded positive results^6^. This has resulted in a growing burden of drug development and is leading researchers to explore alternative approaches. In this context, drug repurposing is considered a promising strategy for identifying new clinical applications for existing drugs^7^. This approach involves screening existing compounds from a database to identify drugs that could be used for dementia treatment. Certain pharmaceuticals, including antidepressants, antihypertensive drugs, anti-inflammatories, and antidiabetic agents, have been shown to be associated with a reduced risk of dementia and improved cognitive function in people with dementia^8–13^. For example, antihypertensive drugs that act on the renin-angiotensin system are associated with slowing the neurodegenerative progression from MCI to AD^9,14^. A systematic review and meta-analysis by Song et al. investigated whether the use of statins could reduce the risk of developing dementia^15^. However, the findings are conflicting, with some studies suggesting a potential neuroprotective effect of statins, while others report acute memory loss in the first thirty days following exposure to statin lipid-lowering drugs^16^, which could be due to detection bias^17^. In contrast, a systematic review by Mejias-Trueba et al. found no significant benefit of statin treatment for patients with dementia^18,19^. Similarly, randomized and placebo-controlled studies assessing the effects of selective serotonin reuptake inhibitors (SSRI) treatment on cognitive function in dementia have produced mixed results^11,20–22^. Some studies suggest that long-term SSRI treatment may delay the progression from mild cognitive impairment (MCI) to dementia, while others found no improvement in cognition performance. Finally, few prospective studies have suggested that protein-pump inhibitor (PPI) use is associated with an increased risk of dementia^23,24^. However, such findings have been inconsistent^25^. Further research is essential to investigate the effects of these drugs and drug classes for dementia and gain a better understanding of the underlying mechanisms of their effects on cognitive function.

To determine the effectiveness of a particular treatment, randomized clinical trials (RCTs) are considered the gold standard. However, conducting large-scale RCTs can be expensive and time-consuming. Therefore, routinely collected observational medical data can be an alternative source of information to evaluate treatment effectiveness. Although the use of electronic health records (EHRs) to investigate the effects of different pharmaceutical drugs on dementia risk and progression shows promise, current research in this area is limited. For example, Olmastroni et al.^18^ found no evidence of neurocognitive risk associated with statin treatment and suggest a potential beneficial effect of statins on cognitive function using observational data. Additional RCTs with an ad hoc design are needed to explore this potential effect of statins further. In addition, most studies have focused on assessing the effects of one drug class, often with conflicting results. To address these limitations, this study aimed to address the current knowledge gap by examining whether some commonly used drugs were associated with a reduced risk of disease progression in individuals with MCI towards dementia.

In this study, we conducted a retrospective cohort study using EHRs from a cohort of 2 million patients seen at a large, multi-specialty, urban academic medical center in New York City, USA. We followed protocols of RCT design^26,27^ and analyzed data from patients who were diagnosed with MCI. Our findings suggest that several drug classes and specific drugs are associated with a delayed diagnosis of dementia in patients with MCI. In particular, we found that the use of beta-blocking agents (BBAs), dihydropyridine derivatives, SSRIs, and specific drugs such as rosuvastatin, citalopram, escitalopram, and omeprazole were associated with reduced risk of progression to dementia. These findings suggest that the repurposing of commonly used drugs may have potential for the prevention or delay of dementia onset in individuals with MCI. However, further RCTs are needed to confirm these findings and to investigate the underlying mechanisms. Additionally, the potential risks and benefits of using these drugs for the treatment of dementia should be carefully evaluated in future studies.

## Methods

### Data source and study cohorts

**Figure 1:**
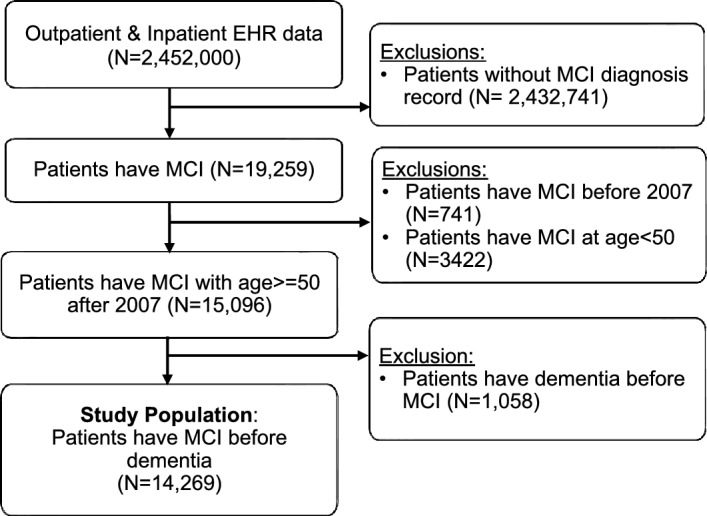
Cascade flow of identifying MCI patients from EHR data.

Using EHR data rendered in the Observational Medical Outcomes Partnership (OMOP) common data model, we conducted a retrospective cohort study from January 1, 2008, to December 31, 2020, on 2,452,000 individuals with both outpatient and inpatient encounters at a major academic medical center in New York City, USA. To be eligible for the study, patients had to meet two criteria: (1) a clinical diagnosis of MCI between January 1, 2008, and December 31, 2020; and (2) be 50 years or older at the time of MCI diagnosis. We identified MCI patients using ICD codes (ICD 9: 331.83, 780.93, ICD10: G31.84, R41.3). Within this group of MCI patients, those with a clinical diagnosis of dementia were identified using ICD codes (ICD 9: 331.0, 294.10, 290.-, ICD10: F02.80, G30.-, F01.-, F02.-, F03). We excluded the all patients with a diagnosis code for dementia prior to MCI diagnosis (Fig. 1).

**Figure 2:**
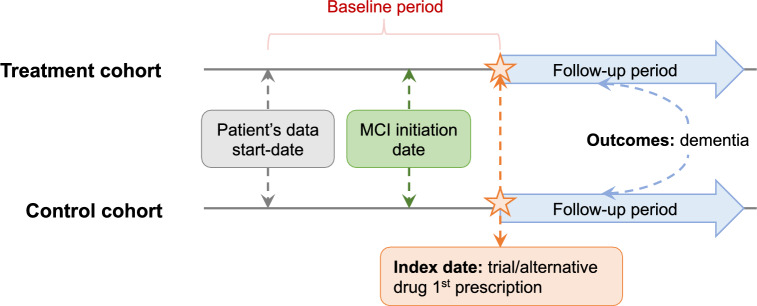
The definition of treatment cohort, control cohort and key dates.

We identified two exposure groups among the MCI patients based on medication status following their MCI diagnosis: the treatment cohort and the control cohort. The treatment cohort consisted of individuals who have been prescribed the study drugs of interest (i.e., trial drugs), while the control cohort comprised individuals using alternative drugs. To establish these groups, we used the Anatomical Therapeutic Chemical (ATC) classification system, as in previous studies^26,27^. This system categorizes medications according to their active ingredients and therapeutic indications. Specifically, we used the second-level ATC class for each drug, denoted as ATC-L2, which includes drugs of the same therapeutic indication. For the alternative treatment (i.e., control cohort), we selected drugs from an ATC class of the trial drug but excluded the drug itself. For drugs already in the second-level ATC class, we used the first-level ATC class for that drug. For example, for ACE-inhibitors (ATC code: C09A), we excluded C09A and used “C09” to identify the control cohort. For beta-blockers (ATC code: C07) whose ATC code is already at the second level, we excluded C07 and used “C” to identify the control cohort. We also excluded patients who were prescribed the trial drug from the control cohort. All patients in both groups were required to take the study drugs after the diagnosis of MCI but before the diagnosis of dementia. To illustrate the process of creating the treatment and control cohorts from the study population, we have included a diagram in Fig. 2. Overall, this approach allows us to investigate the potential effects of specific medications on the progression of dementia in individuals with MCI, while controlling for alternative treatments that may have similar therapeutic indications.

### Study design

We defined the index date as the first (ever) observed prescription date for the specified drug or its alternative medication, as illustrated in Fig. 2. The index date marked the beginning of the treatment or alternative medication period, and we used it as a reference point of our study. We used the observation time prior to the index date as the baseline period and extracted confounding variables during this time. The follow-up period started after the index date and ended when either the onset of dementia was first recorded for patients diagnosed with dementia or when any diagnosis code was last recorded for subjects without dementia. In this study, we defined the MCI initiation date as the first date on which MCI was clinically diagnosed based on ICD-9/10 diagnosis codes.

Our primary outcome for this analysis was the incidence of dementia diagnosis among different medication user groups. During the follow-up period, we measured the efficacy of the medications in terms of the outcome. We hypothesized that several factors could confound the relationship between medication use and the progression of disease from MCI to dementia, including demographics (e.g., age at the index date and gender), comorbidities, prescribed medications, and the time duration from MCI to the first observed prescription date of the study drug. To preprocess these potential confounders, we mapped diagnosis codes to Chronic Conditions Data Warehouse (CCW) algorithms defined by the Centers for Medicare & Medicaid Services (CMS)^28^, and drug prescription codes (i.e., NDC/RxNorm) to the third level of ATC^29^. We assigned one or zero to each feature using a one-hot encoding technique^30^, based on whether patients received a diagnosis or medication in each category or not. We then concatenated all feature variables to represent each patient. As confounders may influence treatment assignment, we calculated all potential confounding variables over the baseline period. Overall, our approach allows us to investigate the potential effects of specific medications while controlling for potential confounding factors among individuals with a diagnosis of MCI.

### Adjust confounding variables using IPTW^31^

To account for potential confounding variables in our analysis, we used the logistic regression-based inverse probability of treatment weighting (IPTW) method^31^. This approach involves assigning a weight to each observation in the data based on the inverse probability of receiving the treatment given the patient's observed characteristics, such that the distribution of covariates is balanced between the treatment and control groups. We evaluated the performance of the model by measuring the feature balance between the weighted treatment and control sub-cohorts generated by IPTW.

To quantify the degree of balance achieved by IPTW, we used the standardized mean difference (SMD) to assess the distribution of each feature between the treatment and control groups before and after weighting^32^. A value of SMD greater than 0.2 indicates that the feature is unbalanced between the groups. We then calculated the ratio of unbalanced features before and after weighting to evaluate the performance of IPTW in balancing the covariates. A low unbalanced feature ratio after weighting indicates that the treatment and control cohorts are well-balanced, with a ratio less than 2% being considered acceptable. This step is to minimize confounding bias and generate a well-balanced treatment and control cohort, making our analysis more robust and reliable.

### Estimate treatment effects of drugs on dementia risk

After balancing the cohorts using IPTW, we investigated the causal effect of drugs on dementia risk by measuring the treatment effects that increased (or decreased) dementia risk. To estimate the treatment effect, we compared the outcomes (i.e., have dementia diagnosis or not) of the treatment group with those of the control group. To mitigate the outcome of this absence, we calculated the average treatment effect (ATE), which is the difference between the expected outcomes of the treatment and control groups. Specifically, ATE = E[Y(1)] − E[Y(0)],^26,27,32^ where E[Y(1)] is the potential outcomes of the treatment group and E[Y(0)] is the potential outcome of the control group. ATE < 0 indicates that the drug is associated with delaying the disease progression from MCI to dementia.

We used the bootstrap method to calculate the confidence intervals of E[Y(1)] and E[Y(0)], as well as the statistical significance of ATE^26,27,33^. The confidence intervals provided a range of values that could reasonably contain the true ATE with a certain level of probability, while the statistical significance indicated whether the ATE was significantly different from zero at a pre-specified level of significance. We resampled a single dataset (95%) via random sampling with replacement to create multiple simulated cohorts (100 iterations). For drugs that did not balance after the first 100 iterations, we performed up to 200 iterations. The total number of balanced trials and iterations are reported in Tables 2 and 3.

### Ethical approval

The study has been approved and the requirement to obtain any informed consent has been waived by the Weill Cornell Medicine Institutional Review Board (protocol no. IRB1408015423). The research does not involve greater than minimal risk for participation. Analyses only involve the secondary analysis of data that are either limited data sets or de-identified. Our research team has no direct contact with human subjects. All methods were carried out in accordance with relevant guidelines and regulations.

## Results

We identified a total of 14,269 patients with MCI who met the inclusion criteria. Out of these patients, 1,247 (8.7%) progressed to AD, and 2501 (17.5%) patients progressed to dementia of any kind, respectively during the study period. The average time to progress from MCI to dementia was 742 days. The characteristics of the study cohort are presented in Table 1. The table shows that the average age of dementia patients in the study cohort (74.0 years old) is higher than that of MCI patients (69.9 years old). Additionally, the study cohort was with more females than males in both the MCI and dementia cohorts. The data also show that almost half of the cohort was composed of white patients, particularly among dementia patients.

**Table 1:** Characteristics of the study cohort.

Characteristics	MCI (n = 14,269)	Progressed to dementia (n = 2501)
Age, Mean (SD), years	69.9 (9.3)	74.0 (7.3)
Sex, No. (%)		
Female	8170 (57.2)	1409 (56.3)
Race, N (%)		
White	6649 (46.6)	1250 (50.0)
Black	1130 (7.9)	211 (8.4)
Asian	460 (3.2)	68 (2.7)
Others	2320 (16.3)	382 (15.3)
Unknown	3710 (26.0)	590 (23.9)
Conversion time, days	511 (MCI lasts)	742

In this study, we evaluated the potential effects of several commonly used drug classes on the progression of dementia. The drug classes that were assessed included antihypertensive drugs, statins, SSRIs, and proton-pump inhibitors (PPIs) (see Supplementary Table S1 for more details). For the antihypertensive drug class, we tested several specific drugs, including ACE-inhibitors, angiotensin receptor blockers (ARBs), beta-blockers, calcium channel blockers (CCBs), diuretics, and fixed combination therapies.

Weighted Kaplan–Meier plots were generated to show the results before and after using a logistic regression (LR) model to reweight the data. The results of these plots are presented in Supplementary Figs. S1–S3. In addition, we report the statistics on the average treatment effects in Table 2. Finally, Table 3 provides the results of the drug ingredients that may be clinically relevant in slowing the progression of dementia.

**Table 2:** The estimated treatment effects for dementia over balanced drug classes.

Drug class	ATC codes	# BT	# Users	# Non-users	*UR (%)	mean ATE (95% CI)	Significance (p-value)
			After reweighting				
Diuretics	C03	83	1366	1729	1.79	0.0328 (0.0255, 0.0401)	< 0.001
Low-ceiling diuretics, thiazides	C03A	100	1327	474	1.78	0.0268 (0.0198, 0.0339)	< 0.001
Beta blockers	C07	100	2661	2631	1.73	0.0221 (0.0141, 0.0300)	< 0.001
BBAs	C07A	100	1404	494	1.10	− 0.0266 (− 0.0341, − 0.019)	< 0.001
BBAs and thiazides	C07B	100	1532	1012	1.55	0.0221 (0.0150, 0.0291)	< 0.001
BBAs and other diuretics	C07C	100	1583	1081	1.73	0.0341 (0.0288, 0.0395)	< 0.001
BBAs, other combinations	C07F	100	1946	697	1.11	0.0147 (0.0075, 0.022)	< 0.001
CCBs	C08	100	719	1083	1.75	0.0211 (0.0138, 0.0284)	< 0.001
Selective CCBs with mainly vascular effects	C08C	100	743	246	1.06	− 0.0458 (− 0.0593, − 0.0324)	< 0.001
Dihydropyridine derivatives	C08CA	100	745	248	1.05	− 0.0571 (− 0.0692, − 0.0451)	< 0.001
Agents acting on the renin-angiotensin system	C09	93	856	1140	1.68	0.0270 (0.0201, 0.0338)	< 0.001
ACE-inhibitors	C09A	100	1145	1544	1.72	0.0341 (0.0283, 0.0399)	< 0.001
ACE inhibitors, combine	C09B	100	1279	1589	1.25	0.1205 (0.1131, 0.1279)	< 0.001
ARBs	C09C	100	1267	1435	1.24	0.0020 (− 0.0037, − 0.0077)	ns
ARBs, combinations	C09D	100	1326	1538	1.19	0.0974 (0.0899, 0.1049)	< 0.001
Statin	C10AA	100	2948	1001	0.98	0.0592 (0.0543, 0.0641)	< 0.001
SSRIs	N06AB	100	1897	2658	1.63	− 0.0210 (− 0.0259, − 0.0162)	< 0.001
PPIs	A02BC	100	2139	758	1.71	0.0274 (0.0224, 0.0324)	< 0.001

*BT:balanced trials; *UR:unbalanced ratio, *ns*=non-significant.

**Table 3:** The estimated treatment effects for dementia over balanced drugs.

Drug name	ATC codes	# BT	# Users	# Non-users	*UR (%)	Mean ATE (95% CI)	Significance
			After reweighting				
Antihypertensive drugs							
Ramipril	C09AA05	6	166	520	1.86	− 0.0388 (− 0.0606, − 0.0169)	ns
losartan	C09CA01	100	902	1723	1.31	− 0.0059 (− 0.011, − 0.0007)	ns
Statins							
Rosuvastatin	C10AA07	100	828	2898	1.28	− 0.0140 (− 0.0191, − 0.0088)	< 0.001
SSRIs							
Citalopram	N06AB04	73	280	1050	1.65	− 0.1128 (− 0.125, − 0.1005)	< 0.001
Escitalopram	N06AB10	100	745	3014	1.60	− 0.0560 (− 0.0615, − 0.0506)	< 0.001
PPIs							
Omeprazole	A02BC01	41	632	1183	1.61	− 0.0201 (− 0.0299, − 0.0103)	< 0.001

### Antihypertensive drugs

The results showed that certain types of antihypertensive drugs were associated with a reduced risk of disease progression to dementia. Specifically, for BBAs, selective CCBs with mainly vascular effects and one type of CCBs—dihydropyridine derivatives, demonstrated a statistically significant association in reducing the risk of progression to dementia. The ATEs with 95% CI for these medications were − 0.0266 (− 0.0341, − 0.019), − 0.0458 (− 0.0593, − 0.0324), and − 0.0571 (− 0.0692, − 0.0451), respectively (Table 2). However, the associations for ARBs were not as clear. The ATE for ARBs was − 0.0048 with 95% CI = [− 0.0078, − 0.0017] (Supplementary Table S1), indicating a potential protective effect in reducing the risk of progression to dementia. However, after adjusting for confounding factors by using IPTW, the protective effect was no longer observed (i.e., ATE was higher than 0). The ATEs for ramipril and losartan were also less than zero, the associations were not statistically significant.

### Statins

The results showed that only rosuvastatin had a statistically significant association with a reduced risk of progression to dementia. The ATE (by IPTW) for rosuvastatin was − 0.0140 with 95% CI = [− 0.0191, − 0.0088] (Table 3), indicating a potential protective effect in reducing the risk of dementia. However, no such effect was observed for other commonly prescribed statins, such as simvastatin, pravastatin, or atorvastatin (Supplementary Table S1).

### SSRIs

The ATEs (by IPTW) for citalopram and escitalopram were − 0.1128 with 95% CI = [− 0.125, − 0.1005] and − 0.0560 with 95% CI = [− 0.0615, − 0.0506] (Table 3), respectively, indicating a potential protective effect in reducing the risk of progression to dementia.

### PPIs

The results showed that omeprazole had a statistically significant association in reducing the risk of progression to dementia. The ATE (by IPTW) for omeprazole was − 0.0201 with 95% CI = [− 0.0299, − 0.0103], indicating a potential protective effect in reducing the risk of dementia. However, for other commonly prescribed PPIs in our cohort, such as lansoprazole, dexlansoprazole, rabeprazole, pantoprazole, and esomeprazole, we were unable to achieve balance using IPTW, as indicated by SMD values greater than 0.2. As a result, we have not included their findings in our report.

## Discussion

The primary aim of this study was to examine the potential protective effect of commonly prescribed drugs in delaying the progression from MCI to dementia, using EHRs as a source of data. To achieve this objective, we implemented a RCT design protocol that enabled us to estimate the causal effect of drugs on dementia risk using observational medical data. By employing robust methods, we were able to adjust for potential confounding factors, evaluate the balancing performance of the treatment and control groups, estimate the ATE and its statistical significance, and calculate the confidence intervals of the treatment effect. The use of these methods allowed us to obtain valuable insights into the effectiveness of drugs in delaying the progression from MCI to dementia. Our findings demonstrated that certain commonly prescribed drug classes and drugs, including antihypertensive agents (beta-blocking agents and dihydropyridine derivatives), statins (rosuvastatin), SSRIs (citalopram and escitalopram), and PPIs (omeprazole), might offer a protective effect in delaying dementia progression. However, not all statins or SSRIs were associated with slowing down the disease progression, suggesting that the observed benefit might be due to specific pharmacokinetic or pharmacodynamic properties of the individual drugs themselves, rather than the treatment of a modifiable risk factor.

The renin-angiotensin system is a target for antihypertensive drugs that have been linked to a reduced rate of neurodegenerative progression from MCI to dementia^11,20^. In our study, we observed that beta-blocking agents (ATC = C07A, ATE [95% CI] = − 0.0266 [− 0.0341, − 0.019]), and dihydropyridine derivatives (ATC = C08CA, ATE [95% CI] =  − 0.0571 [− 0.0692, − 0.0451]) demonstrated a protective effect in reducing the risk of dementia among patients with MCI. Similarly, our study observed that rosuvastatin also demonstrated a protective effect. It is important to note that not all statins or antihypertensive drugs were associated with delayed dementia diagnosis, indicating that the observed advantage is likely due to the unique pharmacokinetic and pharmacodynamic characteristics of each medication. SSRIs are commonly used to treat depression and have been evaluated in randomized, placebo-controlled studies for their effects on cognitive function in Alzheimer’s dementia. The results of these studies have been mixed, with some showing favorable effects^33,34^, no effects^34,35^, or even disadvantageous effects of SSRI treatment^35^. However, our study found that commonly used SSRIs, such as citalopram and escitalopram, had a protective effect in reducing the risk of dementia in MCI patients^36^. This suggests that the impact of SSRIs on dementia risk may be influenced by other factors, such as patient characteristics or the stage of disease progression. PPIs are widely used to treat acid-related diseases, but some prospective studies have suggested that their use may be associated with an increased risk of dementia^25^. However, the evidence regarding this association is inconsistent^25^. In our study, we did not observe a protective effect in reducing the risk of dementia in MCI patients who took PPIs, with the exception of omeprazole, which demonstrates a statistically significant protective effect. Further research is needed to determine the exact relationship between PPI use and dementia risk, as well as to identify the underlying mechanisms involved.

This study has several limitations that need to be acknowledged. First, the use of ICD diagnosis codes instead of formal clinical diagnostic criteria to identify patients with MCI and dementia is a significant limitation. While ICD codes are widely used to identify patients with a particular diagnosis, they are not as reliable as formal clinical diagnostic criteria. There is a risk of misclassification or misdiagnosis if relying solely on ICD codes. Second, this study did not consider drug dosages, which could impact the estimated ATEs. The differences in ATEs between higher and lower drug dosages must be investigated to understand the optimal dosage that could maximize the protective effects of these drugs. Third, medication adherence and prescription refills were not accounted for due to the lack of prescription fill data. Therefore, it is challenging to determine the extent of medication adherence and the frequency of prescription refills for each patient. Fourth, the results of this study were not replicated using an external EHR data set. This study cohort was predominantly derived from patients at a large, urban academic medical center in New York City, and it may not fully represent the general population across the United States. Additionally, there may be different environmental and social factors associated with urban settings. Replicating this study using external EHR data sets would be an important next step to validate these findings. Finally, the limitations of this study extend to the nature of dementia itself. Dementia reflects progressive neurodegeneration that usually takes decades to develop. The dataset derived from EHRs may not fully capture the medical history across the lifespan for each study participant. Additionally, the cohort matching approach may lead to bias, as mismatched patients are excluded from the analysis. Moreover, patients often have mixed dementia, such as AD plus vascular dementia and/or dementia with Lewy bodies, and the heterogeneity of AD itself is manifested through its complex pathobiology and may be related to genetic background, environmental factors, and other causal triggers^4^. These measures are not typically incorporated into routine clinical practice, limiting the scope of the study.

## Conclusion

This study contributes to the literature on evaluating the protective effects of commonly prescribed drugs in reducing the risk of dementia among MCI patients. To the best of our knowledge, this is one of the early studies that utilized EHR data obtained during routine clinical encounters to examine the protective effects of such treatments. The results of this study are partially consistent with previous findings reported in the literature, although further validation is necessary to confirm the observed associations and explore the underlying mechanisms. The study demonstrates that despite the limitation of real-world data, such as incomplete or inaccurate data, the analysis of drug effectiveness using real-world data can be a valuable tool in supporting future drug repurposing studies.

## Supplementary Information

Supplementary Information.

## Data Availability

All data required to evaluate the conclusions of the manuscript are presented in the main text and/or the Supplementary Materials. The dataset used during the current study is a HIPAA limited data set, which requires a data use agreement, https://its.weill.cornell.edu/services/research-informatics/ehr-data-and-reporting. Request of the data can be sent to NYP’s External Data Use and Sharing (EDUS) committee.
