# Supplementary material for: Comparing the effects of four common drug classes on the progression of mild cognitive impairment to dementia using electronic health records
Source: Sci Rep. 2023 May 19;13:8102. doi: 10.1038/s41598-023-35258-6 (PMC10199021; doi:10.1038/s41598-023-35258-6)
Supplement: Supplementary file 1 — Supplementary Information. [file 41598_2023_35258_MOESM1_ESM.docx]

**Supplementary Information: Comparing the Effects of Four Common Drug Classes on the Progression of Mild Cognitive Impairment to Dementia Using Electronic Health Records**

Jie Xu, PhD^1,2^, Fei Wang, PhD^2^, Chengxi Zang, PhD^2^, Hao Zhang, PhD^2^, Kellyann Niotis, MD^2^, Ava L. Liberman, MD^2^, Cynthia M. Stonnington, MD^3^, Makoto Ishii, MD, PhD^2^, Prakash Adekkanattu^2^, Yuan Luo, PhD^4^, Chengsheng Mao, PhD^4^, Luke V Rasmussen, PhD^4^, Zhenxing Xu, PhD^2^, Pascal Brandt, PhD^5^, Jennifer A Pacheco, PhD^4^, Yifan Peng, PhD^2^, Guoqian Jiang, MD, PhD^6^, Richard Isaacson, MD^2^, Jyotishman Pathak, PhD^2,*^

^1^University of Florida, Gainesville, FL, USA

^2^Weill Cornell Medicine, Cornell University, New York, NY, USA

^3^Mayo Clinic, Scottsdale, Arizona, USA

^4^Feinberg School of Medicine, Northwestern University, Chicago, IL, USA

^5^University of Washington, Seattle, Washington, USA

^6^Mayo Clinic, Rochester, Minnesota, USA

*Corresponding Author

#### **Table S1**. The Estimated Treatment Effects for dementia over Balanced Drugs/Drug Classes (Extended Table 2 in the main manuscript, sorted by “ATC codes”).

|  | **Drug Class/Drug** | **ATC**  **Codes** | **# BT** | **# Users** | **# Non-**  **users** | ***UR (%)** | **mean ATE (95% CI)** | |
| --- | --- | --- | --- | --- | --- | --- | --- | --- |
|  |  |  |  | **After Reweighting** | | | | |
|  | Proton pump inhibitors | A02BC | 100 | 2139 | 758 | 1.71 | 0.0274 | (0.0224, 0.0324) |
|  | omeprazole; systemic | A02BC01 | 41 | 632 | 1183 | 1.61 | -0.0201 | (-0.0299, -0.0103) |
|  | ANTIHYPERTENSIVES AND DIURETICS IN COMBINATION | C02L | 100 | 1161 | 381 | 1.09 | 0.0469 | (0.0381, 0.0556) |
|  | Rauwolfia alkaloids and diuretics in combination | C02LA | 100 | 1477 | 478 | 0.61 | 0.0617 | (0.0546, 0.0688) |
|  | reserpine and diuretics, combinations with psycholeptics | C02LA71 | 100 | 1474 | 477 | 0.58 | 0.0708 | (0.063, 0.0786) |
|  | Hydrazinophthalazine derivatives and diuretics | C02LG | 100 | 1226 | 402 | 1.19 | 0.0412 | (0.0333, 0.0492) |
|  | picodralazine and diuretics, combinations with psycholeptics | C02LG73 | 100 | 1474 | 476 | 0.62 | 0.0663 | (0.0589, 0.0737) |
|  | DIURETICS | C03 | 83 | 1366 | 1729 | 1.79 | 0.0328 | (0.0255, 0.0401) |
|  | LOW-CEILING DIURETICS, THIAZIDES | C03A | 100 | 1327 | 474 | 1.78 | 0.0268 | (0.0198, 0.0339) |
|  | Thiazides, combinations with other drugs | C03AX | 67 | 1667 | 753 | 1.67 | 0.0432 | (0.0364, 0.05) |
|  | hydrochlorothiazide, combinations | C03AX01 | 73 | 1691 | 759 | 1.73 | 0.038 | (0.0329, 0.0431) |
|  | BETA BLOCKING AGENTS | C07 | 100 | 1064 | 1860 | 1.73 | 0.0221 | (0.0141, 0.03) |
|  | BETA BLOCKING AGENTS | C07A | 100 | 1404 | 494 | 1.10 | -0.0266 | (-0.0341, -0.019) |
|  | Beta blocking agents, selective | C07AB | 100 | 1303 | 1044 | 1.42 | 0.0076 | (0.0009, 0.0143) |
|  | metoprolol; systemic | C07AB02 | 100 | 842 | 980 | 1.65 | 0.0275 | (0.0196, 0.0353) |
|  | BETA BLOCKING AGENTS AND THIAZIDES | C07B | 100 | 1520 | 1008 | 1.46 | 0.0233 | (0.0173, 0.0294) |
|  | Beta blocking agents, selective, and thiazides | C07BB | 100 | 1149 | 1265 | 1.57 | 0.0308 | (0.0232, 0.0384) |
|  | metoprolol and thiazides | C07BB02 | 100 | 999 | 1231 | 1.58 | 0.0224 | (0.0164, 0.0284) |
|  | metoprolol and thiazides, combinations | C07BB52 | 100 | 1005 | 1239 | 1.62 | 0.0178 | (0.0115, 0.0241) |
|  | BETA BLOCKING AGENTS AND OTHER DIURETICS | C07C | 100 | 1583 | 1081 | 1.73 | 0.0341 | (0.0288, 0.0395) |
|  | Beta blocking agents, selective, and other diuretics | C07CB | 100 | 1390 | 1200 | 1.72 | 0.0297 | (0.0228, 0.0367) |
|  | metoprolol and other diuretics | C07CB02 | 100 | 1002 | 1064 | 1.66 | 0.026 | (0.0194, 0.0326) |
|  | BETA BLOCKING AGENTS, OTHER COMBINATIONS | C07F | 100 | 1946 | 697 | 1.11 | 0.0147 | (0.0075, 0.022) |
|  | Beta blocking agents and calcium channel blockers | C07FB | 100 | 1219 | 1038 | 1.52 | 0.0368 | (0.0311, 0.0424) |
|  | metoprolol and felodipine | C07FB02 | 100 | 851 | 1152 | 1.55 | 0.0283 | (0.0216, 0.0351) |
|  | metoprolol and amlodipine | C07FB13 | 100 | 988 | 1027 | 1.58 | 0.0286 | (0.0211, 0.0361) |
|  | Beta blocking agents, other combinations | C07FX | 100 | 1764 | 869 | 1.38 | 0.0407 | (0.0354, 0.046) |
|  | metoprolol and acetylsalicylic acid | C07FX03 | 100 | 1599 | 973 | 1.35 | 0.0662 | (0.0597, 0.0728) |
|  | metoprolol and ivabradine | C07FX05 | 100 | 810 | 1132 | 1.60 | 0.0213 | (0.0148, 0.0277) |
|  | CALCIUM CHANNEL BLOCKERS | C08 | 100 | 719 | 1083 | 1.75 | 0.0211 | (0.0138, 0.0284) |
|  | SELECTIVE CALCIUM CHANNEL BLOCKERS WITH MAINLY VASCULAR EFFECTS | C08C | 100 | 743 | 246 | 1.06 | -0.0458 | (-0.0593, -0.0324) |
|  | Dihydropyridine derivatives | C08CA | 100 | 745 | 248 | 1.05 | -0.0571 | (-0.0692, -0.0451) |
|  | nifedipine, combinations | C08CA55 |  | 1707 | 1081 | 1.43 | 0.0101 | (0.0037, 0.0164) |
|  | AGENTS ACTING ON THE RENIN-ANGIOTENSIN SYSTEM | C09 | 93 | 856 | 1140 | 1.68 | 0.027 | (0.0201, 0.0338) |
|  | ACE INHIBITORS, PLAIN | C09A | 100 | 1146 | 1543 | 1.71 | 0.0345 | (0.0284, 0.0406) |
|  | ACE inhibitors, plain | C09AA | 100 | 1145 | 1544 | 1.72 | 0.0341 | (0.0283, 0.0399) |
|  | lisinopril; oral | C09AA03 | 100 | 737 | 1901 | 1.66 | 0.064 | (0.0581, 0.0698) |
|  | ramipril; oral | C09AA05 | 6 | 166 | 520 | 1.86 | -0.0388 | (-0.0606, -0.0169) |
|  | ACE INHIBITORS, COMBINATIONS | C09B | 100 | 1279 | 1589 | 1.25 | 0.1205 | (0.1131, 0.1279) |
|  | ACE inhibitors and diuretics | C09BA | 100 | 1027 | 1816 | 0.95 | 0.1104 | (0.1037, 0.1171) |
|  | captopril and diuretics | C09BA01 | 69 | 777 | 2043 | 1.44 | 0.0557 | (0.0431, 0.0684) |
|  | enalapril and diuretics | C09BA02 | 97 | 816 | 2011 | 1.48 | 0.0324 | (0.0223, 0.0425) |
|  | lisinopril and diuretics | C09BA03 | 100 | 973 | 1874 | 1.00 | 0.0992 | (0.0905, 0.108) |
|  | perindopril and diuretics | C09BA04 | 67 | 768 | 2051 | 1.61 | 0.0426 | (0.0312, 0.054) |
|  | ramipril and diuretics | C09BA05 | 70 | 790 | 2034 | 1.55 | 0.0522 | (0.0417, 0.0627) |
|  | quinapril and diuretics | C09BA06 | 43 | 771 | 2050 | 1.60 | 0.0503 | (0.0348, 0.0657) |
|  | benazepril and diuretics; oral | C09BA07 | 59 | 773 | 2052 | 1.49 | 0.0498 | (0.037, 0.0625) |
|  | cilazapril and diuretics | C09BA08 | 66 | 768 | 2051 | 1.50 | 0.0342 | (0.0209, 0.0475) |
|  | fosinopril and diuretics | C09BA09 | 66 | 773 | 2052 | 1.51 | 0.0471 | (0.0365, 0.0578) |
|  | delapril and diuretics | C09BA12 | 71 | 772 | 2053 | 1.44 | 0.0548 | (0.0442, 0.0653) |
|  | moexipril and diuretics | C09BA13 | 65 | 772 | 2053 | 1.43 | 0.0377 | (0.0247, 0.0507) |
|  | zofenopril and diuretics | C09BA15 | 58 | 773 | 2053 | 1.47 | 0.0489 | (0.0377, 0.0602) |
|  | ANGIOTENSIN II RECEPTOR BLOCKERS (ARBs), PLAIN | C09C | 100 | 1267 | 1435 | 1.24 | 0.002 | (-0.0037, 0.0077) |
|  | Angiotensin II receptor blockers (ARBs), plain | C09CA | 100 | 1265 | 1436 | 1.26 | 0.0029 | (-0.0039, 0.0096) |
|  | losartan; oral | C09CA01 | 100 | 902 | 1723 | 1.31 | -0.0059 | (-0.011, -0.0007) |
|  | valsartan; oral | C09CA03 | 100 | 214 | 726 | 1.61 | 0.083 | (0.0739, 0.092) |
|  | ANGIOTENSIN II RECEPTOR BLOCKERS (ARBs), COMBINATIONS | C09D | 100 | 1326 | 1538 | 1.19 | 0.0974 | (0.0899, 0.1049) |
|  | Angiotensin II receptor blockers (ARBs) and diuretics | C09DA | 100 | 1125 | 1753 | 1.12 | 0.0789 | (0.0724, 0.0854) |
|  | losartan and diuretics | C09DA01 | 100 | 992 | 1874 | 1.34 | 0.0244 | (0.0165, 0.0324) |
|  | eprosartan and diuretics | C09DA02 | 66 | 770 | 2052 | 1.49 | 0.0572 | (0.0485, 0.0659) |
|  | valsartan and diuretics | C09DA03 | 100 | 867 | 1969 | 1.34 | 0.1062 | (0.0972, 0.1151) |
|  | irbesartan and diuretics | C09DA04 | 70 | 787 | 2030 | 1.48 | 0.0892 | (0.0774, 0.101) |
|  | candesartan and diuretics | C09DA06 | 69 | 771 | 2052 | 1.54 | 0.0429 | (0.0316, 0.0543) |
|  | telmisartan and diuretics; oral | C09DA07 | 96 | 787 | 2035 | 1.45 | 0.0601 | (0.0512, 0.069) |
|  | olmesartan medoxomil and diuretics | C09DA08 | 77 | 795 | 2029 | 1.45 | 0.0811 | (0.0716, 0.0906) |
|  | azilsartan medoxomil and diuretics | C09DA09 | 65 | 772 | 2053 | 1.43 | 0.0437 | (0.0326, 0.0548) |
|  | fimasartan and diuretics | C09DA10 | 62 | 769 | 2050 | 1.47 | 0.0567 | (0.0463, 0.0671) |
|  | HMG CoA reductase inhibitors | C10AA | 100 | 2948 | 1001 | 0.98 | 0.0592 | (0.0543, 0.0641) |
|  | simvastatin; oral | C10AA01 | 100 | 875 | 3036 | 1.11 | 0.0496 | (0.0441, 0.0551) |
|  | pravastatin; oral | C10AA03 | 100 | 295 | 1144 | 1.44 | 0.1391 | (0.1327, 0.1455) |
|  | atorvastatin; oral | C10AA05 | 100 | 2036 | 2267 | 1.53 | 0.0394 | (0.0334, 0.0455) |
|  | rosuvastatin; oral | C10AA07 | 100 | 828 | 2896 | 1.28 | -0.014 | (-0.0191, -0.0088) |
|  | Selective serotonin reuptake inhibitors | N06AB | 100 | 1897 | 2658 | 1.63 | -0.021 | (-0.0259, -0.0162) |
|  | citalopram; systemic | N06AB04 | 73 | 280 | 1050 | 1.65 | -0.1128 | (-0.125, -0.1005) |
|  | sertraline; oral | N06AB06 | 100 | 395 | 1719 | 1.58 | 0.0206 | (0.0123, 0.0289) |
|  | escitalopram; oral | N06AB10 | 100 | 745 | 3014 | 1.60 | -0.056 | (-0.0615, -0.0506) |

*BT: ​​Balanced Trials; *UR: Unbalanced Ratio

#### **Table S2**. The Estimated Treatment Effects for dementia over Drug Classes/Drugs (before reweighting, sorted by “ATC codes”).

|  | **Drug Class/Drug** | **ATC**  **Codes** | **# Users** | **# Non-**  **users** | ***UR (%)** | **mean ATE (95% CI)** | |
| --- | --- | --- | --- | --- | --- | --- | --- |
|  |  |  | **Before Reweighting** | | | | |
|  | Proton pump inhibitors | A02BC | 2618 | 804 | 12.59 | 0.0235 | (0.0208, 0.0261) |
|  | omeprazole; systemic | A02BC01 | 1337 | 2200 | 40.57 | -0.0134 | (-0.02, -0.0068) |
|  | ANTIHYPERTENSIVES AND DIURETICS IN COMBINATION | C02L | 1738 | 399 | 34.95 | 0.0146 | (0.0109, 0.0182) |
|  | Rauwolfia alkaloids and diuretics in combination | C02LA | 1617 | 513 | 32.53 | 0.0276 | (0.0243, 0.0309) |
|  | reserpine and diuretics, combinations with psycholeptics | C02LA71 | 1617 | 513 | 32.20 | 0.0317 | (0.0287, 0.0347) |
|  | Hydrazinophthalazine derivatives and diuretics | C02LG | 1714 | 421 | 36.26 | 0.0153 | (0.0124, 0.0183) |
|  | picodralazine and diuretics, combinations with psycholeptics | C02LG73 | 1617 | 513 | 32.48 | 0.0301 | (0.0269, 0.0334) |
|  | DIURETICS | C03 | 2897 | 2406 | 42.10 | 0.0176 | (0.0117, 0.0234) |
|  | LOW-CEILING DIURETICS, THIAZIDES | C03A | 2465 | 522 | 11.83 | 0.0407 | (0.037, 0.0443) |
|  | Thiazides, combinations with other drugs | C03AX | 2221 | 872 | 12.62 | 0.0548 | (0.0513, 0.0584) |
|  | hydrochlorothiazide, combinations | C03AX01 | 2221 | 872 | 12.50 | 0.0524 | (0.0491, 0.0557) |
|  | BETA BLOCKING AGENTS | C07 | 2661 | 2631 | 43.30 | 0.0275 | (0.0225, 0.0325) |
|  | BETA BLOCKING AGENTS | C07A | 2238 | 502 | 34.31 | 0.0271 | (0.0239, 0.0303) |
|  | Beta blocking agents, selective | C07AB | 1634 | 1118 | 8.58 | 0.0433 | (0.0397, 0.0469) |
|  | metoprolol; systemic | C07AB02 | 1386 | 1409 | 11.80 | 0.0435 | (0.0393, 0.0477) |
|  | BETA BLOCKING AGENTS AND THIAZIDES | C07B | 1761 | 1065 | 17.77 | 0.0223 | (0.0194, 0.0253) |
|  | Beta blocking agents, selective, and thiazides | C07BB | 1457 | 1364 | 15.04 | 0.0354 | (0.0311, 0.0397) |
|  | metoprolol and thiazides | C07BB02 | 1420 | 1398 | 18.06 | 0.0321 | (0.0285, 0.0357) |
|  | metoprolol and thiazides, combinations | C07BB52 | 1420 | 1398 | 17.96 | 0.0291 | (0.0257, 0.0326) |
|  | BETA BLOCKING AGENTS AND OTHER DIURETICS | C07C | 1721 | 1103 | 33.31 | 0.0131 | (0.0105, 0.0158) |
|  | Beta blocking agents, selective, and other diuretics | C07CB | 1578 | 1243 | 31.16 | 0.0222 | (0.019, 0.0254) |
|  | metoprolol and other diuretics | C07CB02 | 1551 | 1268 | 36.15 | 0.0261 | (0.0219, 0.0303) |
|  | BETA BLOCKING AGENTS, OTHER COMBINATIONS | C07F | 2085 | 718 | 26.43 | 0.032 | (0.0294, 0.0345) |
|  | Beta blocking agents and calcium channel blockers | C07FB | 1640 | 1171 | 23.17 | 0.035 | (0.0317, 0.0384) |
|  | metoprolol and felodipine | C07FB02 | 1336 | 1468 | 17.17 | 0.0353 | (0.0319, 0.0387) |
|  | metoprolol and amlodipine | C07FB13 | 1578 | 1243 | 29.64 | 0.0329 | (0.0285, 0.0373) |
|  | Beta blocking agents, other combinations | C07FX | 1919 | 903 | 22.94 | 0.028 | (0.0257, 0.0304) |
|  | metoprolol and acetylsalicylic acid | C07FX03 | 1785 | 1029 | 27.96 | 0.0406 | (0.0375, 0.0438) |
|  | metoprolol and ivabradine | C07FX05 | 1327 | 1478 | 17.91 | 0.0322 | (0.0285, 0.0359) |
|  | CALCIUM CHANNEL BLOCKERS | C08 | 2797 | 2557 | 44.11 | 0.0274 | (0.0211, 0.0337) |
|  | SELECTIVE CALCIUM CHANNEL BLOCKERS WITH MAINLY VASCULAR EFFECTS | C08C | 2588 | 260 | 16.18 | 0.0355 | (0.0315, 0.0396) |
|  | Dihydropyridine derivatives | C08CA | 2588 | 260 | 16.76 | 0.0356 | (0.0319, 0.0393) |
|  | nifedipine, combinations | C08CA55 | 1834 | 1102 | 26.89 | 0.0255 | (0.0224, 0.0285) |
|  | AGENTS ACTING ON THE RENIN-ANGIOTENSIN SYSTEM | C09 | 2776 | 2530 | 43.92 | 0.0338 | (0.0283, 0.0393) |
|  | ACE INHIBITORS, PLAIN | C09A | 1212 | 1637 | 5.70 | 0.013 | (0.0099, 0.0162) |
|  | ACE inhibitors, plain | C09AA | 1212 | 1637 | 5.74 | 0.012 | (0.0092, 0.0149) |
|  | lisinopril; oral | C09AA03 | 841 | 2054 | 36.12 | 0.0132 | (0.0104, 0.0159) |
|  | ramipril; oral | C09AA05 | 184 | 2598 | 9.49 | -0.0153 | (-0.0317, 0.0011) |
|  | ACE INHIBITORS, COMBINATIONS | C09B | 1312 | 1699 | 63.03 | 0.0205 | (0.0178, 0.0233) |
|  | ACE inhibitors and diuretics | C09BA | 1058 | 1938 | 62.84 | 0.0189 | (0.0162, 0.0216) |
|  | captopril and diuretics | C09BA01 | 802 | 2171 | 66.12 | 0.0052 | (0.0021, 0.0084) |
|  | enalapril and diuretics | C09BA02 | 843 | 2139 | 65.71 | 0.0023 | (-0.0003, 0.0049) |
|  | lisinopril and diuretics | C09BA03 | 1000 | 1997 | 64.47 | 0.0156 | (0.0126, 0.0187) |
|  | perindopril and diuretics | C09BA04 | 794 | 2179 | 65.99 | 0.0058 | (0.0026, 0.0089) |
|  | ramipril and diuretics | C09BA05 | 812 | 2159 | 65.07 | 0.0107 | (0.0072, 0.0143) |
|  | quinapril and diuretics | C09BA06 | 792 | 2176 | 65.87 | 0.0056 | (0.0011, 0.0101) |
|  | benazepril and diuretics; oral | C09BA07 | 794 | 2177 | 66.04 | 0.0075 | (0.0037, 0.0112) |
|  | cilazapril and diuretics | C09BA08 | 794 | 2179 | 66.05 | 0.0061 | (0.003, 0.0093) |
|  | fosinopril and diuretics | C09BA09 | 795 | 2178 | 66.09 | 0.0038 | (0.0009, 0.0067) |
|  | delapril and diuretics | C09BA12 | 794 | 2179 | 66.05 | 0.0052 | (0.0021, 0.0084) |
|  | moexipril and diuretics | C09BA13 | 794 | 2179 | 65.87 | 0.0017 | (-0.0019, 0.0052) |
|  | zofenopril and diuretics | C09BA15 | 794 | 2179 | 66.16 | 0.0066 | (0.0027, 0.0105) |
|  | ANGIOTENSIN II RECEPTOR BLOCKERS (ARBs), PLAIN | C09C | 1414 | 1537 | 4.39 | -0.0048 | (-0.0078, -0.0017) |
|  | Angiotensin II receptor blockers (ARBs), plain | C09CA | 1414 | 1537 | 4.35 | -0.0041 | (-0.0076, -0.0007) |
|  | losartan; oral | C09CA01 | 1091 | 1891 | 9.76 | -0.021 | (-0.0237, -0.0184) |
|  | valsartan; oral | C09CA03 | 298 | 2535 | 11.83 | 0.022 | (0.0174, 0.0266) |
|  | ANGIOTENSIN II RECEPTOR BLOCKERS (ARBs), COMBINATIONS | C09D | 1355 | 1643 | 55.86 | 0.0146 | (0.0112, 0.0179) |
|  | Angiotensin II receptor blockers (ARBs) and diuretics | C09DA | 1145 | 1863 | 55.40 | 0.0095 | (0.0068, 0.0123) |
|  | losartan and diuretics | C09DA01 | 1017 | 1996 | 60.85 | -0.0077 | (-0.0106, -0.0048) |
|  | eprosartan and diuretics | C09DA02 | 794 | 2179 | 65.98 | 0.0065 | (0.003, 0.0099) |
|  | valsartan and diuretics | C09DA03 | 889 | 2093 | 62.77 | 0.0151 | (0.0119, 0.0184) |
|  | irbesartan and diuretics | C09DA04 | 814 | 2160 | 65.10 | 0.0134 | (0.01, 0.0168) |
|  | candesartan and diuretics | C09DA06 | 794 | 2178 | 65.94 | 0.0074 | (0.004, 0.0108) |
|  | telmisartan and diuretics; oral | C09DA07 | 814 | 2163 | 65.26 | 0.0013 | (-0.0013, 0.004) |
|  | olmesartan medoxomil and diuretics | C09DA08 | 818 | 2154 | 65.52 | 0.0097 | (0.0071, 0.0124) |
|  | azilsartan medoxomil and diuretics | C09DA09 | 794 | 2179 | 66.24 | 0.0053 | (0.002, 0.0087) |
|  | fimasartan and diuretics | C09DA10 | 794 | 2179 | 66.06 | 0.0064 | (0.0033, 0.0094) |
|  | HMG CoA reductase inhibitors | C10AA | 3611 | 1006 | 4.31 | 0.0646 | (0.0622, 0.067) |
|  | simvastatin; oral | C10AA01 | 1023 | 3480 | 32.30 | 0.0245 | (0.0225, 0.0265) |
|  | pravastatin; oral | C10AA03 | 412 | 4083 | 57.66 | 0.0221 | (0.0188, 0.0253) |
|  | atorvastatin; oral | C10AA05 | 2418 | 2373 | 41.12 | 0.0153 | (0.0123, 0.0182) |
|  | rosuvastatin; oral | C10AA07 | 992 | 3636 | 42.69 | -0.0142 | (-0.0167, -0.0117) |
|  | Selective serotonin reuptake inhibitors | N06AB | 2305 | 2921 | 5.22 | -0.0286 | (-0.031, -0.0261) |
|  | citalopram; systemic | N06AB04 | 474 | 4628 | 7.38 | -0.0754 | (-0.0813, -0.0696) |
|  | sertraline; oral | N06AB06 | 716 | 4431 | 35.94 | 0.0023 | (-0.0021, 0.0066) |
|  | escitalopram; oral | N06AB10 | 1119 | 4094 | 13.56 | -0.0536 | (-0.0564, -0.0508) |

*UR: Unbalanced Ratio

## **Weighted Kaplan–Meier survival curves.**

Survival curves are estimated for each group, considered separately, using the weighted Kaplan-Meier method. Figures S1-S3 present the Weighted Kaplan–Meier survival curves and associated 95% CIs for the three drug classes. After adjusting the confounders, MCI patients on SSRIs had significantly delayed the progression to dementia compared to patients on other drugs.

####
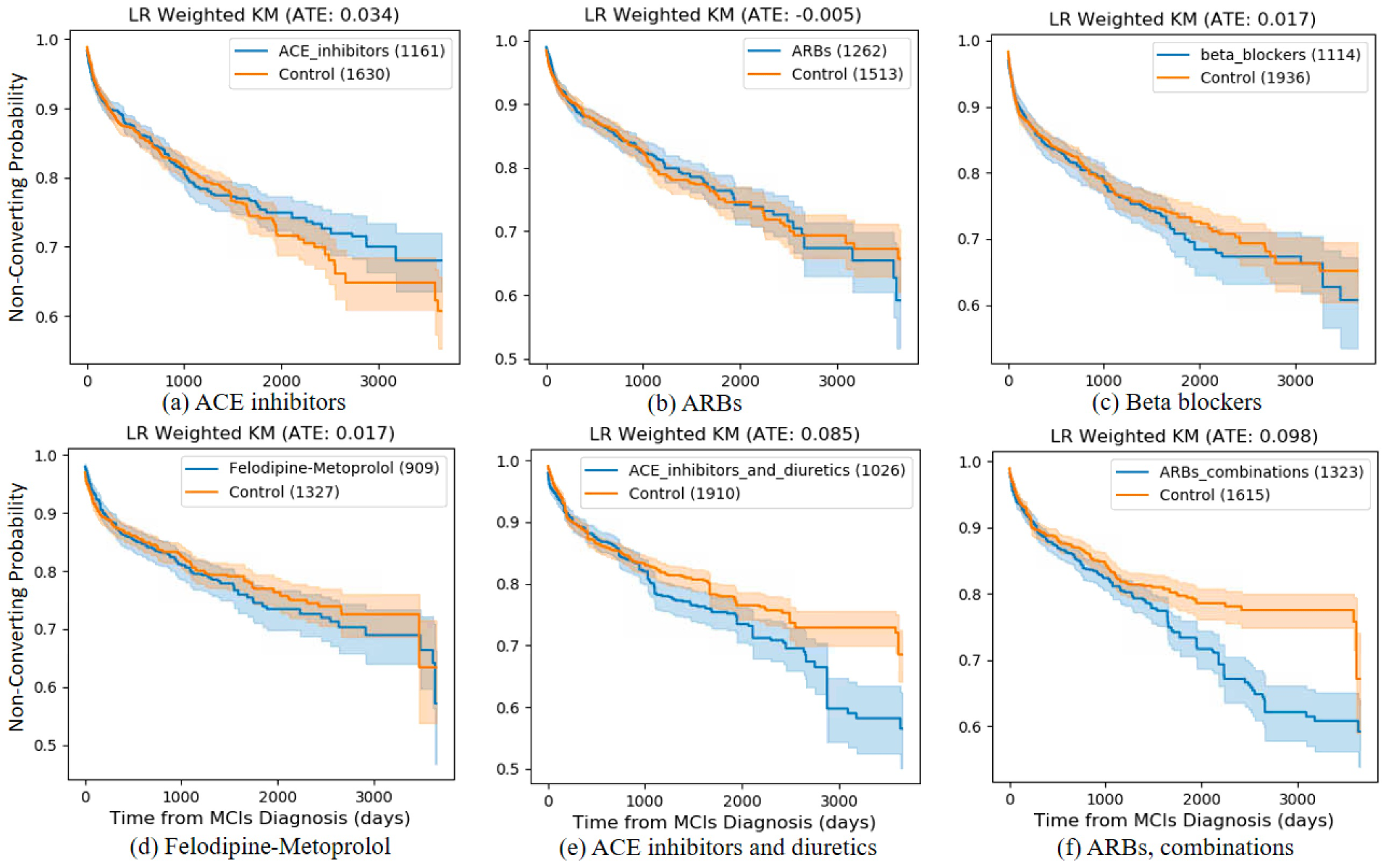


#### **Figure S1**. Weighted Kaplan-Meier Plots of antihypertensive. Illustration of the LR model for predicting treatment probability that is used to adjust confounding from data.

####
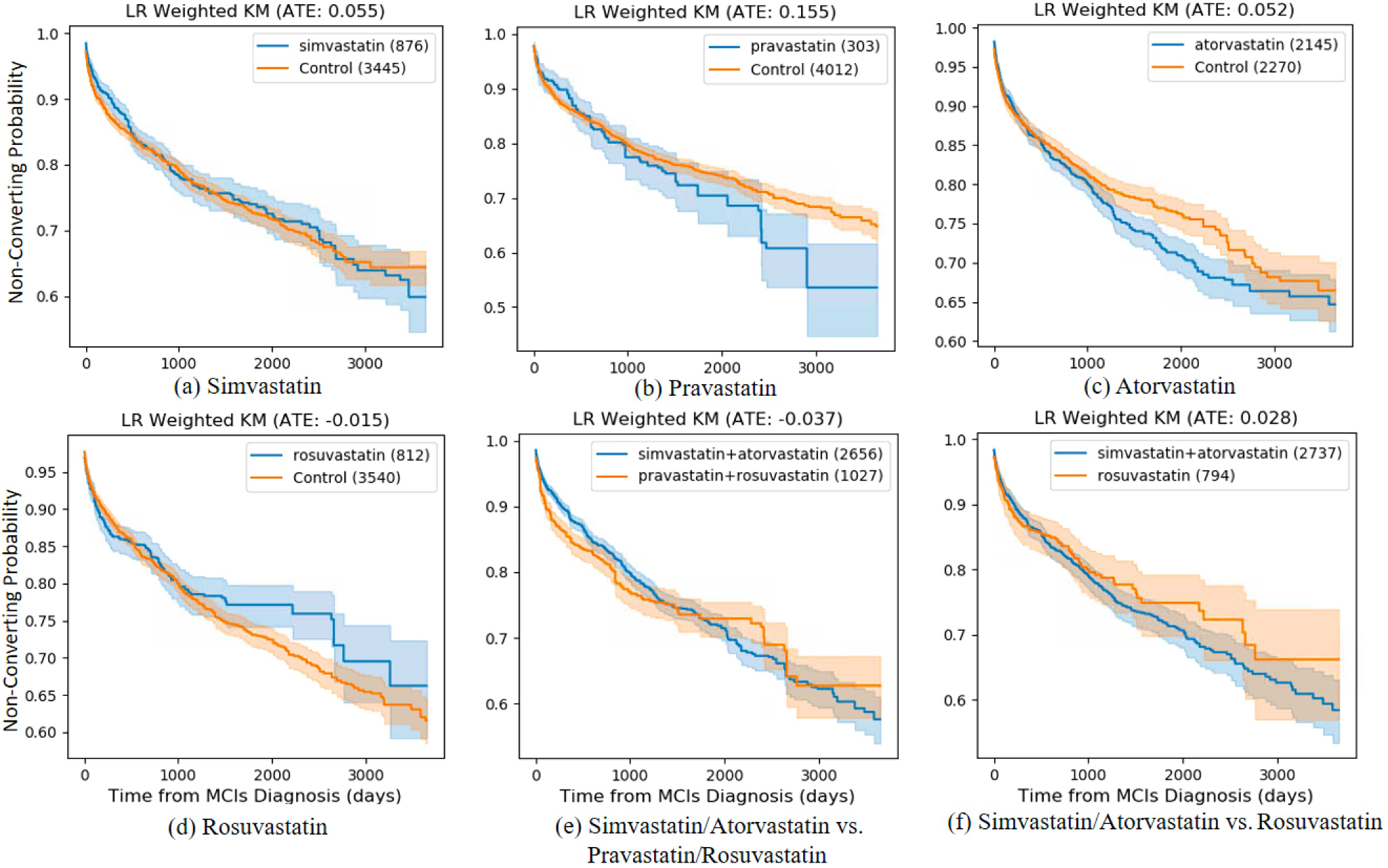


#### **Figure S2**. Weighted Kaplan-Meier Plots of Statins. Illustration of the LR model for predicting treatment probability that is used to adjust confounding from data.


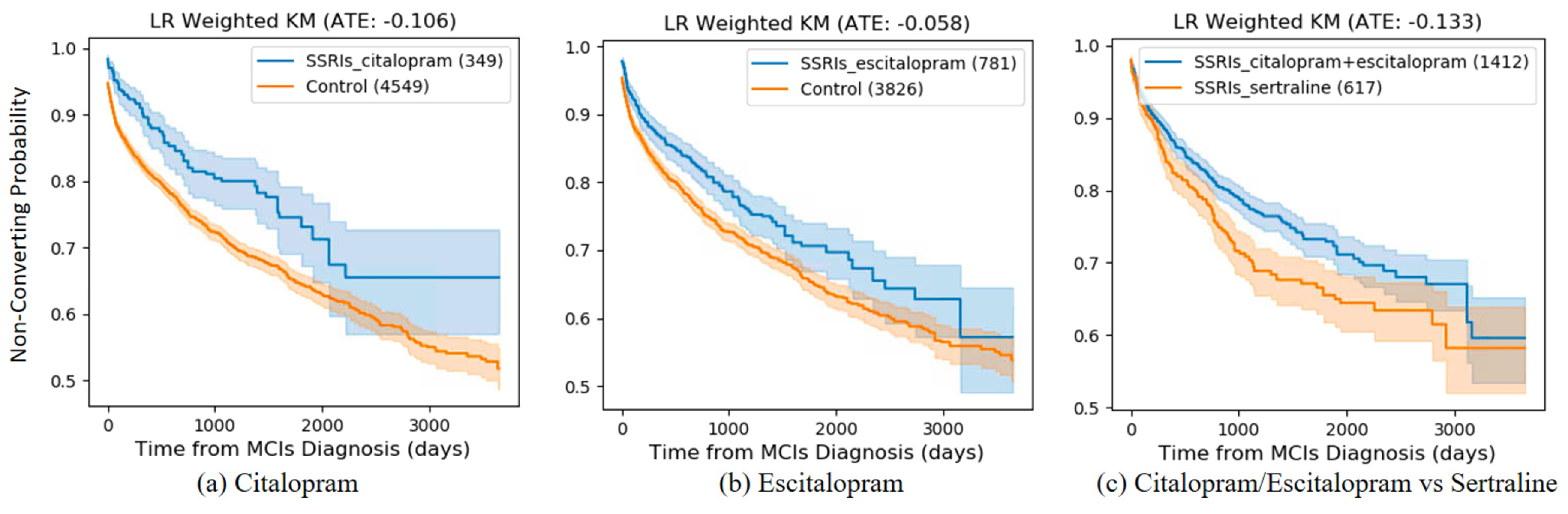


#### **Figure S3**. Weighted Kaplan-Meier Plots of SSRIs. Illustration of the LR model for predicting treatment probability that is used to adjust confounding from data.
